# Effects of plant extracts on patients with heart failure: a network meta-analysis of randomized controlled trials

**DOI:** 10.3389/fphar.2025.1648811

**Published:** 2025-11-05

**Authors:** Jieyin Deng, Ye Huang, Dehui Fu, Yi Deng, Ke Yu, Cong Lan

**Affiliations:** ^1^ Department of General Medical Practice, The General Hospital of Western Theater Command, Chengdu, China; ^2^ Department of Nursing, Nursing School, Chengdu Medical College, Chengdu, China; ^3^ Department of Cardiology, The General Hospital of Western Theater Command, Chengdu, China

**Keywords:** heart failure, plant extract, network meta-analysis, Chinese patent medicine, conventional therapy

## Abstract

**Ethnopharmacological relevance:**

Heart failure (HF) is a major global health challenge. Traditional therapies have limitations, while recent studies highlight plant extracts’ potential due to their medicinal properties and milder side effects.

**Objective:**

This study conducts a systematic review and network meta-analysis (NMA) to assess the therapeutic effects of plant extracts on patients with heart failure, providing robust evidence for clinical practice.

**Materials and methods:**

A comprehensive search of databases, including PubMed, Embase, and Cochrane Library, was performed. Studies were screened using predefined criteria to extract data and assess quality. Network meta-analysis enabled direct/indirect comparisons of multiple plant extracts’ efficacy in heart failure intervention.

**Results:**

A total of 20 studies encompassing 2,077 patients were incorporated into the analysis. Astragalus extract demonstrated the highest efficacy in enhancing the 6-min walk test (6-MWT) score (surface under the cumulative ranking curve [SUCRA]: 90.70%) and reducing tumor necrosis factor-alpha (TNF-α) levels (SUCRA: 74.4%). Shenfu extract exhibited superior efficacy in decreasing B-type natriuretic peptide (BNP) values (SUCRA: 68.2%) and enhancing the quality of life (QL) (SUCRA: 77.0%). Red ginseng extract was more effective in improving left ventricular ejection fraction (LVEF) (SUCRA: 77.9%), while *Ginkgo biloba* extract showed greater efficacy in ameliorating New York Heart Association (NYHA) functional classification (SUCRA: 76.5%). Despite these findings, heterogeneity and methodological issues in the studies warrant further high-quality, large-scale randomized controlled trials (RCTs) to validate the results and determine the optimal plant extract use in heart failure treatment.

**Conclusion:**

Astragalus extract, red ginseng extract, *Ginkgo biloba* extract, *Terminalia arjuna* extract, and Shenfu extract have demonstrated significant efficacy in heart failure management in the study for the selection of optimal plant extracts based on enhanced indicators of cardiac function outcomes. Continued research through rigorous randomized controlled trials is essential to substantiate and refine the current evidence.

## 1 Introduction

Heart failure (HF) constitutes a significant and intricate heterogeneous syndrome characterized by a collection of signs and symptoms arising from cardiac dysfunction, including impaired ventricular filling or compromised blood ejection; clinical manifestations such as dyspnea, fatigue, and peripheral and pulmonary edema are commonly observed, contributing to reduced life expectancy. Moreover, the prevalence of heart failure is rising (Teng et al., 2024; Mosterd and Hoes, 2007; McKee et al., 1971; Yancy et al., 2017). The majority of patients experience impaired function, diminished quality of life (QL), and premature mortality as a result of heart failure, a condition that is clinically associated with high morbidity and mortality rates and reduced quality of life and survival (Hao et al., 2015; Trupp, 2007). Heart failure represents a significant and escalating public health challenge, imposing a considerable burden on healthcare systems. There remains a need for the development and enhancement of novel and alternative therapeutic approaches for heart failure (Tao et al., 2023). Cornerstone medications in Western medicine for heart failure include angiotensin-converting enzyme inhibitors/angiotensin receptor blockers (ACEI/ARB), beta-blockers, mineralocorticoid receptor antagonists, and sodium–glucose co-transporter-2 (SGLT2) inhibitors (Heidenreich et al., 2022). Non-pharmacological approaches include cardiac resynchronization therapy (McDonagh et al., 2021). Despite advances in standard therapy, the prognosis for patients with heart failure remains poor, with ongoing challenges in clinical symptom management and quality of life improvement.

Traditional Chinese medicine (TCM) has evolved over more than two millennia, with herbal medicine having a longstanding history as an adjunctive therapy in the management of heart failure (Tsai et al., 2017). In recent decades, TCM has gained increasing popularity (Hao et al., 2015) and has demonstrated efficacy in enhancing the quality of life for patients with heart failure (Tang and Huang, 2013). Shao-Mei et al. (2022) concluded that the TCM approach of benefiting qi is effective in treating heart failure and improving cardiac metabolism, sharing common features with other treatment methods. Chinese medicine is extensively utilized in clinical practice (Han et al., 2017). Wang K. et al. (2017) asserted that the integration of Chinese and Western medical treatments (including injections) is more effective in enhancing the overall clinical efficacy and improving the condition of patients with heart failure. The combination of Chinese herbal medicine and conventional pharmaceuticals in the treatment of heart failure is emerging as a trend in contemporary medicine (Xu et al., 2022).

Tao et al. (2023) found that ginseng injection enhanced clinical efficiency and cardiac function in patients with heart failure. Zheng et al. (2011) reported that Shenmai improved cardiac index and myocardial contractility, while Pittler et al. (2008) noted the significant efficacy of hawthorn extract in managing heart failure symptoms. The proprietary Chinese medicine Qiliqiangxin capsule has shown promise in treating heart failure (Wang Y. et al., 2017), and Xinmailong capsule has been found to be more effective than Western medications for the same condition (Ma et al., 2013). However, previous studies have primarily focused on herbal compound preparations, leaving a gap in scientific evidence regarding the effectiveness of specific herbal monomers in improving cardiac function in patients with heart failure.

Network meta-analysis (NMA) represents an advanced methodological approach that facilitates the ranking of interventions through both direct and indirect comparisons, contrasting with the conventional pairwise meta-analysis (Leucht et al., 2013). In this study, we employed a meta-analytic technique to investigate key Chinese medicine monomers, including hawthorn, Danhong, astragalus, red ginseng, *Terminalia arjuna*, and Shenfu, in the context of heart failure treatment. Our objective was to assess the efficacy and role of these Chinese medicine monomers in managing heart failure, thereby contributing a foundational basis for their clinical application.

## 2 Materials and methods

### 2.1 Search strategy

Our literature search encompassed PubMed, Web of Science, Embase, and Cochrane databases up to September 2024. We conducted a systematic review of 20 studies (patients) through a meta-analysis. The research strategy was built around the PICOS tools: (P) population: patients with heart failure; (I) intervention: plant extracts; (C) comparator: the control group received only the usual treatment for heart failure; (O) outcomes: indicators of improvement in cardiac function, brain natriuretic peptide (BNP), New York Heart Association (NYHA), left ventricular ejection fraction (LVEF), the 6-min walk test (6-MWT), tumor necrosis factor-alpha (TNF-α), and quality of life questionnaire (QLQ; and (S) study types: randomized controlled trials (RCTs). The detailed search strategy is shown in Table 1 (PubMed is used as an example).

**TABLE 1:** PubMed search strategy.

Search	PubMed
#1	Search: Heart failure [MeSH terms] 155056
#2	((((((((((((((Heart Failure) OR (Cardiac Failure)) OR (Heart Decompensation)) OR (Decompensation, Heart)) OR (Congestive Heart Failure)) OR (Heart Failure, Congestive)) OR (Heart Failure, Right-Sided)) OR (Heart Failure, Right Sided)) OR (Right-Sided Heart Failure)) OR (Right Sided Heart Failure)) OR (Heart Failure, Left-Sided)) OR (Heart Failure, Left Sided)) OR (Left-Sided Heart Failure)) OR (Left Sided Heart Failure)) OR (Myocardial Failure) 365787
#3	(#1) OR (#2) 365787
#4	(((((((((((((((((((((((((Astragalosides AS-IV) OR (Salvianolic acid A)) OR (Salvianolic acid B)) OR (Tanshinol)) OR (Salidroside)) OR (Puerarin)) OR (Ginsenosides)) OR (Panax notoginseng saponins)) OR (Safflower Yellower)) OR (Carthamin)) OR (Ferulic acid sodium)) OR (Ligustrazine)) OR (Amygdalin)) OR (Paeoniflorin)) OR (Hawthorn flavonoids)) OR (Tanshinone IIA)) OR (triterpenoid saponin)) OR (Zingiberol)) OR (Ginkgo Biloba Extract, GBE)) OR (Dioscin)) OR (Digitoxin)) OR (Ophiopogon saponin)) OR (Saikoside)) OR (Atractylol)) OR (Aconitine)) OR (Hesperidin) 36820
#5	(#3) AND (#4) 707
#6	Randomized controlled 971464
#7	(#5) AND (#6) 42

### 2.2 Inclusion criteria

The following inclusion criteria were used: (1) an experimental group of heart failure patients treated with different plant extracts; (2) a control group receiving conventional treatment only for patients with heart failure; (3) randomized controlled clinical trials; (4) outcome measures including at least one of the following: brain natriuretic peptide (BNP), heart failure classification (NYHA), LVEF, TNF-α, 6-MWT, and QLQ; and (5) full-text availability.

### 2.3 Exclusion criteria

The following were the exclusion criteria: (1) data missing or unreported in the study and (2) non-observational studies such as clinical randomized controlled trials, randomized trials, animal studies, summaries, reviews, case reports, or letters.

### 2.4 Study selection

The literature was screened and excluded using literature management software EndNote. Researchers screened titles for duplication, non-randomized controlled trials, review papers, conference papers, protocols, and correspondence. Two researchers read the abstracts of the studies to identify which literature should be included or excluded. After this, the remaining literature was read in full by both researchers to determine whether it should be included. A third researcher discussed and resolved the differences between the two researchers’ findings after they both independently screened the literature.

### 2.5 Data extraction

Data from eight categories were recorded and standardized before choosing the data extraction table to include in the study: (1) author, (2) country, (3) year of publication, (4) cardiac functional grading, (5) age, (6) sample size, (7) intervention, and (8) outcome indicator.

### 2.6 Risk of bias of individual studies

Following the Cochrane Handbook version 5.1.0 guidelines for assessing the risk of bias (RoB) in RCTs, two researchers independently reviewed the RoB considering the following seven domains (Higgins et al., 2011): (1) generation of random sequences, (2) concealing treatment allocation, (3) blinding of participants and (4) personnel, (5) incomplete data on outcomes, (6) selective reporting, and (7) additional bias sources. The trials were organized into three levels of RoB by counting the components with the potential for high RoB: high risk (five or more), moderate risk (three or four), and low risk (two or fewer).

### 2.7 Data analysis

In the studies we included that utilized plant extracts as interventions, heart function was assessed using binary variables, while continuous variables, expressed as standard deviation (SD) (Li and Chen, 2021), were utilized for the remaining measures. In this study, continuous variables will be presented as either the mean difference (MD), which represents the absolute difference between the means of the treatment and control groups calculated on the same scale or the standardized mean difference (SMD), which is the mean difference in outcomes between groups divided by the standard deviation of outcomes among subjects. The SMD is used to synthesize data from trials utilizing different scales. Both MD and SMD will be reported with 95% confidence intervals (CIs) and accompanied by appropriate analyses.

As per the instructions in the PRISMA NMA instruction manual, NMA aggregates and analyses were performed using Stata software (version 15.1) with Markov chain Monte Carlo simulation chains within a Bayesian framework (Harrington et al., 2021; Shim et al., 2017). Using the nodal method, we assessed and illustrated the alignment between indirect and direct comparisons, which were determined according to Stata software guidelines, and a *p*-value greater than 0.05 was considered to indicate passing the consistency test (Salanti et al., 2011).

Network diagrams of different plant extracts are presented and described using Stata software. Within these generated network diagrams, each node symbolizes a distinct motor intervention or control condition, while the lines interconnecting the nodes denote direct head-to-head comparisons between the interventions. The dimensions of each node and the thickness of the connecting lines are proportional to the number of studies included (Thom et al., 2019).

P-scores were calculated based on the intervention hierarchy. As a frequentist equivalent to the surface under the cumulative ranking curve (SUCRA), p-scores indicate the likelihood that a treatment is better than any other treatment, averaged across all competitors. P-scores range from 0 to 1, where 1 indicates the best treatment with no uncertainty and 0 indicates the worst treatment with no uncertainty. To assess the potential bias introduced by small-scale studies, which could contribute to publication bias in NMA, a network funnel plot was constructed and examined for symmetry (Harbord et al., 2006).

## 3 Results

### 3.1 Search results and study selection

**FIGURE 1:**
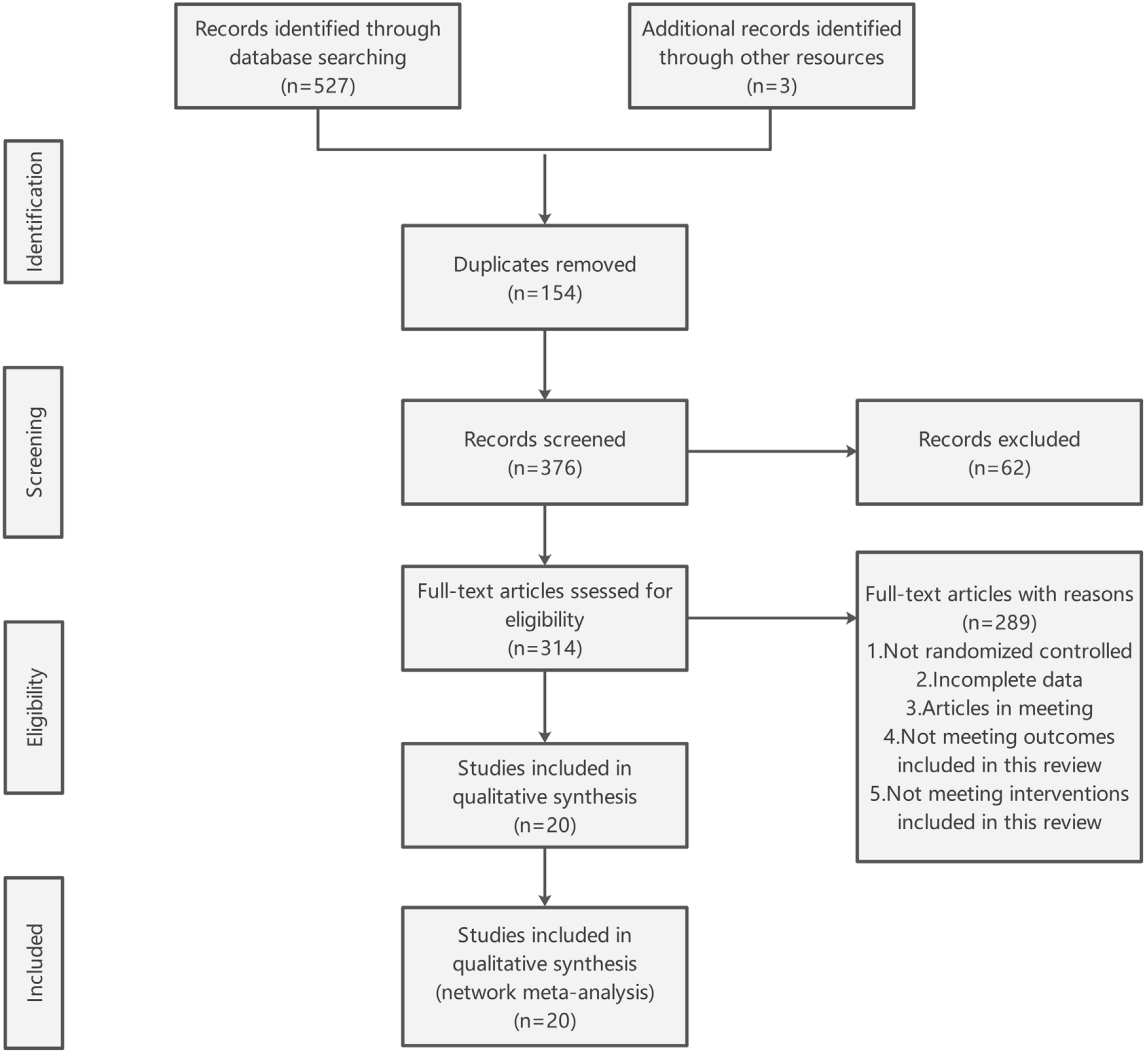
Flow diagram of study selection.

A comprehensive search of the electronic database yielded 527 documents, supplemented by an additional three documents identified through manual searching. Following the removal of duplicates, 376 documents remained for the screening of titles and abstracts, resulting in the exclusion of 62 documents. Subsequently, the full texts of the remaining 314 documents were reviewed, leading to the exclusion of 289 documents due to reasons such as non-randomized controlled trial design, incomplete data, conference paper format, and non-compliance with the interventions specified in this review. Ultimately, 20 documents were deemed suitable for inclusion in this study (Figure 1).

### 3.2 Quality assessment of the included studies

**FIGURE 2:**
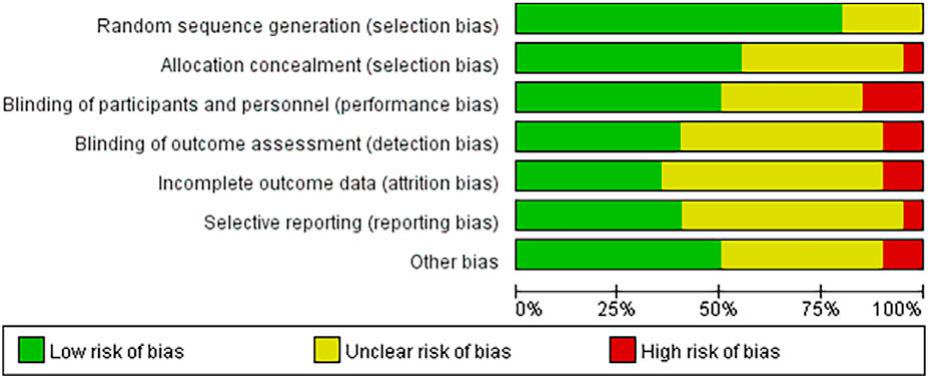
Risk of bias gragh. Bar chart showing risk of bias in various categories: Random sequence generation, Allocation concealment, Blinding of participants and personnel, Blinding of outcome assessment, Incomplete outcome data, Selective reporting, and Other bias. Each category is represented by a colored bar indicating low (green), unclear (yellow), and high (red) risk of bias.

**FIGURE 3:**
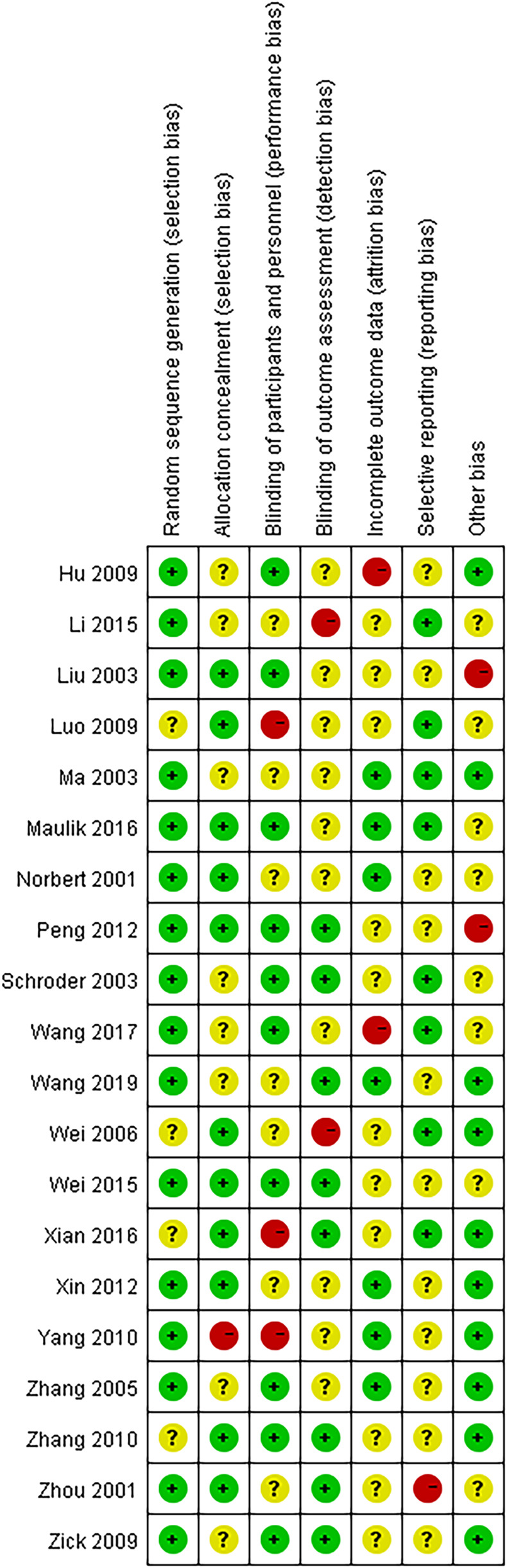
Risk of bias summary. Risk of bias summary chart for multiple studies, showing different biases like random sequence generation, allocation concealment, blinding, etc. Each study, labeled on the left, uses colored circles to indicate risk: green for low, yellow for unclear, and red for high. Columns represent various bias categories.

The assessment of the formation of RoB is presented in Figures 2, 3. The assessment data for the RoB are presented in Table 2. Among the 20 studies included, 2 studies satisfied 2 Cochrane criteria, 7 studies satisfied 3 criteria, 10 studies satisfied 4 criteria, and 1 study satisfied 5 criteria. All studies either possessed complete datasets or provided sufficient explanations and employed appropriate methodologies for addressing the missing data. The potential for other biases was deemed low across all studies. Overall, the majority of the 20 trials were assessed as having relatively moderate risk levels.

**TABLE 2:** Characteristics of the studies included in the meta-analysis.

Author	Country	Year	Cardiac functional grading	Age (mean + SD)	Total/male/female	Intervention	Dosage (day)	Treatment period	Control	Outcome
Maulik	India	2016	T: NR C: NR	T: 40.4 (9.8) C: 45.2 (8.9)	T: 50/31/19 C: 50/30/20	Arjuna (capsules)	750 mg	6 weeks 12 weeks	Placebo	BNP, NYHA, LVEF, 6-MWT, TNF-α, and QLQ
Wei	China	2015	T: NR C: NR	T: 75 (9) C: 69 (9)	T: 18/8/10 C: 22/13/9	Shenfu (granule)	NR	14 d	Con	LVEF, TNF-α, and QLQ
Hu	China	2009	T: 0/0/20/11 C: 0/0/22/10	T: 72.94 (7.58) C: 76.43 (6.80)	T: 31/12/19 C: 32/12/20	Shenfu (injection) Con	40 mL	7 days	Con	LVEF
Luo	China	2009	T: 0/9/32/9 C: 0/10/28/12	T: 59.3 (5.1) C: 63.2 (7.1)	T: 50/3/17 C: 50/28/22	Shenfu (injection) Con	60 mL	10 days each month, total of 6 months	Con	NYHA and LVEF
Li	China	2015	T: NR C: NR	T: 80.03 (5.36) C: 79.21 (5.37)	T: 30/15/15 C: 30/16/14	Shenfu (injection)	200 mL	14 days	Con	BNP, NYHA, and LVEF
Wang	China	2019	T: 0/0/46/48 C: 0/0/51/19	T: 68.58 (8.42) C: 68.14 (8.73)	T: 74/42/32 C: 70/48/32	Shenfu (injection) Con	50 mL	7 days	Con	NYHA, LVEF, and 6-MWT
Xian	China	2016	T: 0/55/50/9 C: 0/56/43/15	T: 68.95 (9.91) C: 68.12 (8.88)	T: 114/71/43 C: 114/66/48	Shenfu (injection)	100 mL	7 days	Con	BNP, NYHA, LVEF, 6-MWT, and QLQ
Yang	China	2010	T: 0/9/13/0 C: 0/8/15/0	T: 63 (4.7) C: 62.5 (4.3)	T: 22/12/10 C: 23/13/10	Astragalus (granule) Con	3 g	14 days	Con	NYHA, LVEF, LVEF, 6-MWT, and TNF-α
Liu	China	2003	T: 0/6/15/10 C: 0/5/14/12	T: 62.7 (7.2) C: 63.1 (7.5)	T: 31/21/10 C: 31/19/12	Astragalus (injection) Con	30 mL	20 days	Con	NYHA and LVEF
Wei	China	2006	T: NR C: NR	T: NR C: NR	T: 25/NR/NR C: 24/NR/NR	Astragalus (injection) Con	40 mL	15 days	Con	LVEF and TNF-α
Zhang	China	2005	T: 0/9/17/10 C: 0/10/16/10	T: 67.3 (8.7) C: 68.2 (9.6)	T: 36/20/16 C: 36/21/15	Astragalus (injection) Con	30 mL	28 days	Con	LVEF and TNF-α
Zhou	China	2001	T: 0/7/23/12 C: 0/6/24/11	T: 68 (5) C: 67 (6)	T: 42/24/18 C: 41/25/16	Astragalus (injection)	40 mL	14 days	Nitroglycerin	NYHA and LVEF
Zick	European	2009	T: 0/30/30/0 C: 0/33/27/0	T: 54.4 (12.6) C: 57.8 (9)	T: 60/46/14 C: 60/44/16	Hawthorn (tablet)	900 mg	3 months 6 months	Placebo	BNP, NYHA, LVEF, 6-MWT, and QLQ
Xin	China	2012	T: 0/5/14/9 C: 0/6/18/4	T: 58.9 (8.7) C: 59.6 (9.2)	T: 28/15/13 C: 28/16/12	Red ginseng (injection) Con	40 mL	7 days	Con	NYHA and LVEF
Wang	China	2017	T: 0/19/86/61 C: 0/17/95/56	T: 67 (9) C: 67 (9)	T: 166/91/75 C: 168/92/76	Danhong + Shenfu (injection) Danhong + Shenmai (injection)	60 mL + 40 mL	7 days	Con	BNP, LVEF, 6-MWT, and QLQ
Schroder	Germany	2003	T: NR C: NR	T: 68.5 (7.85) C: 65.6 (9.06)	T: 110/32/78 C: 102/48/54	Hawthorn (oral drops)	NR	8 weeks	ACE inhibitor diuretics	6-MWT
Norbert	Germany	2001	T: NR C: NR	T: 66.5 (7.8) C: 63 (7.6)	T: 44/14/31 C: 44/24/20	Hawthorn (oral drops)	NR	6 weeks 12 weeks	Placebo	QLQ
Ma	China	2003	T: 0/7/16/7 C: 0/6/16/8	T: 65.27 (1.86) C: 67.33 (1.61)	T: 30/17/13 C: 30/15/15	Red ginseng (injection) Con	100 mL	14 days	Con	NYHA
Peng	China	2012	T: 0/0/11/29 C: 0/0/13/27	T: 63 (12.4) C: 64 (11.7)	T: 40/22/18 C: 40/23/17	Red ginseng (injection)	40 mL	28 days	Con levocarnitine	NYHA
Zhang	China	2010	T: 0/26/15/0 C: 0/24/16/0	T: 57.2 (14.3) C: 58.9 (14.0)	T: 41/25/16 C: 40/27/13	Ginkgo (tablet) Diuretics ACE inhibitor diuretics	2 tablets	24 weeks	Diuretics	NYHA

CON, control group with routine care (no exercise); T, experimental group; C, control group; BNP, brain natriuretic peptide; NYHA, heart failure classification (NYHA); LVEF, left ventricular ejection fraction; TNF-α, tumor necrosis factor-α; 6-MWT, 6-min walk test; QLQ, life quality questionnaire; NR, unreported; Mo, month; w, week; d, day.

### 3.3 Characteristics of the included studies

In total, we included 20 randomized controlled trials encompassing 2,077 patients diagnosed with heart failure. The control group included Shenfu extract (six studies) (Wei et al., 2015; Hu et al., 2009; Luo et al., 2009; Li et al., 2015; Wang X. et al., 2019; Xian et al., 2016), astragalus extract (five studies) (Yang et al., 2010; Liu et al., 2003; Wei et al., 2006; Zhang et al., 2005; Zhou et al., 2001), hawthorn extract (three studies) (Zick et al., 2009; Schröder et al., 2003; Rietbrock et al., 2001), red ginseng extract (three studies) (Xin and Shan, 2012; Ma et al., 2003; Peng and Zhao, 2012), *Ginkgo biloba* extract (one study) (Zhang et al., 2010), *Terminalia arjuna* extracts (one study) (Maulik et al., 2016), and Danhong extract (one study) (Wang X. et al., 2017). One study was from India, one was from Europe, two were from Germany, and 16 were from China. The characteristics of the included studies are shown in Table 2.

### 3.4 Network meta-analysis

**FIGURE 4:**
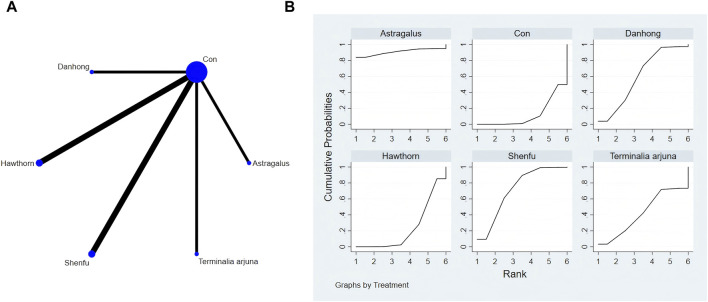
**(A)** NMA figure for 6-MWT. **(B)** SUCRA plot for 6-MWT.

**FIGURE 5:**
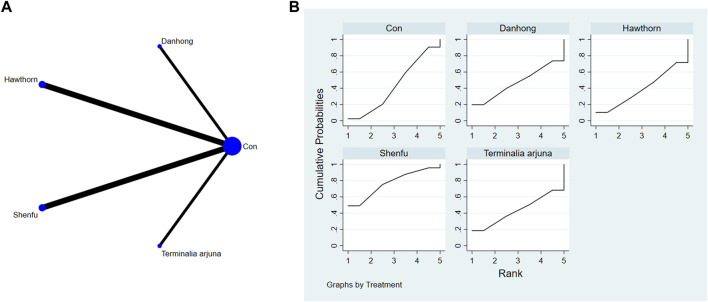
**(A)** NMA figure for BNP. **(B)** SUCRA plot for BNP.

**FIGURE 6:**
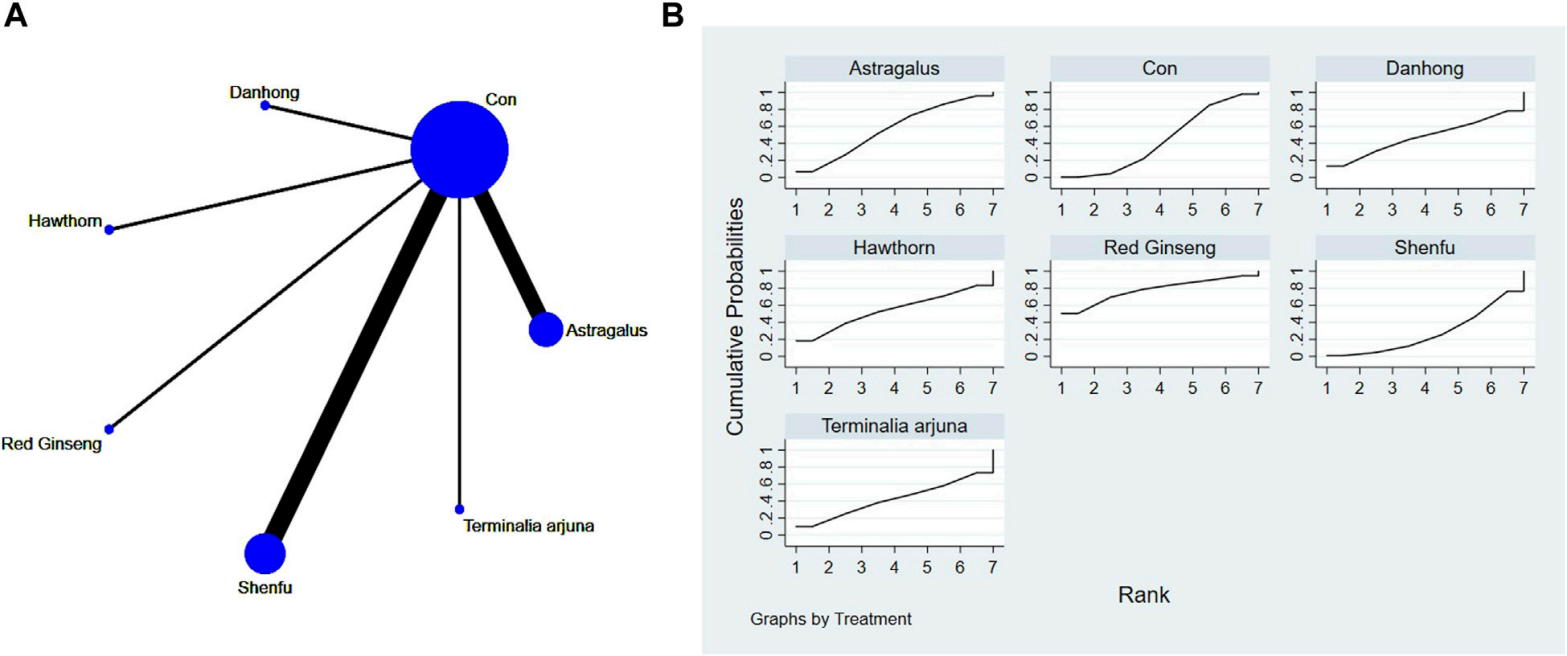
**(A)** NMA figure for LVEF. **(B)** SUCRA plot for LVEF.

**FIGURE 7:**
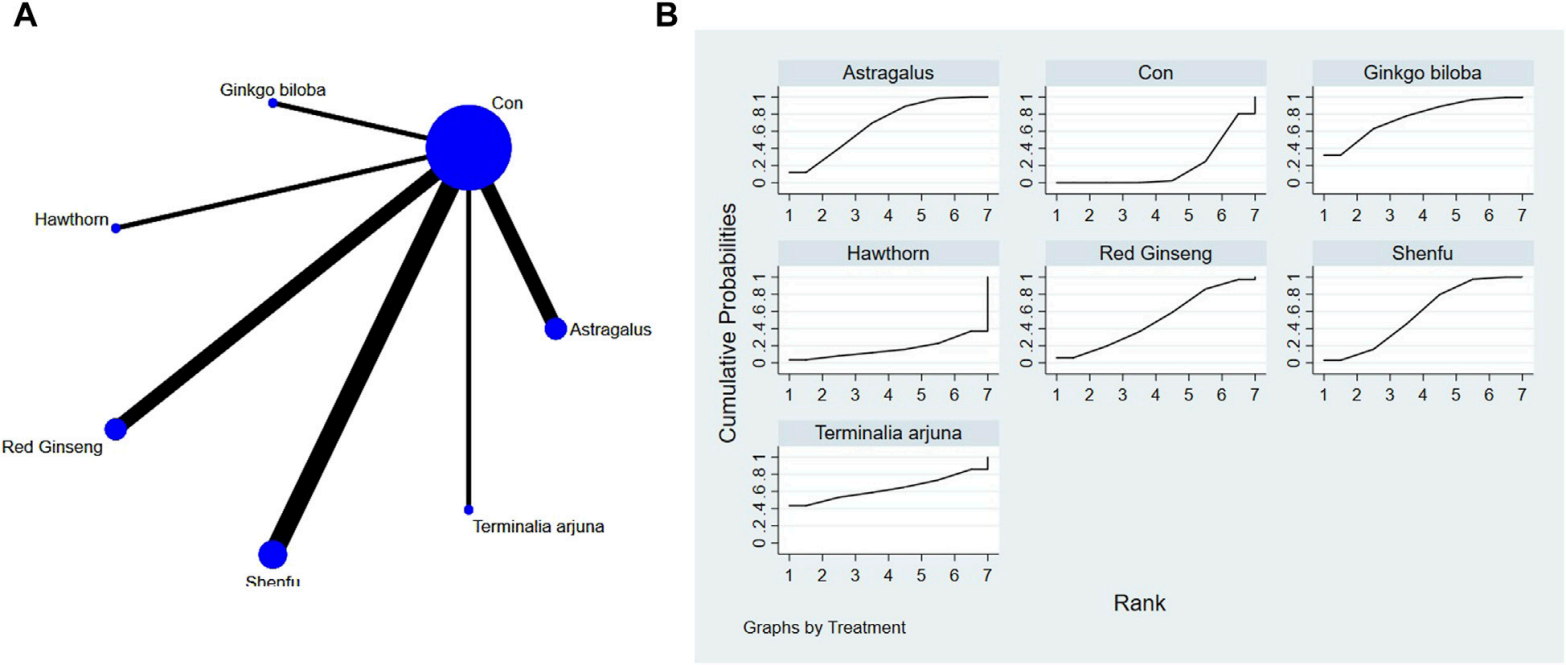
**(A)** NMA figure for NYHA. **(B)** SUCRA plot for NYHA.

**FIGURE 8:**
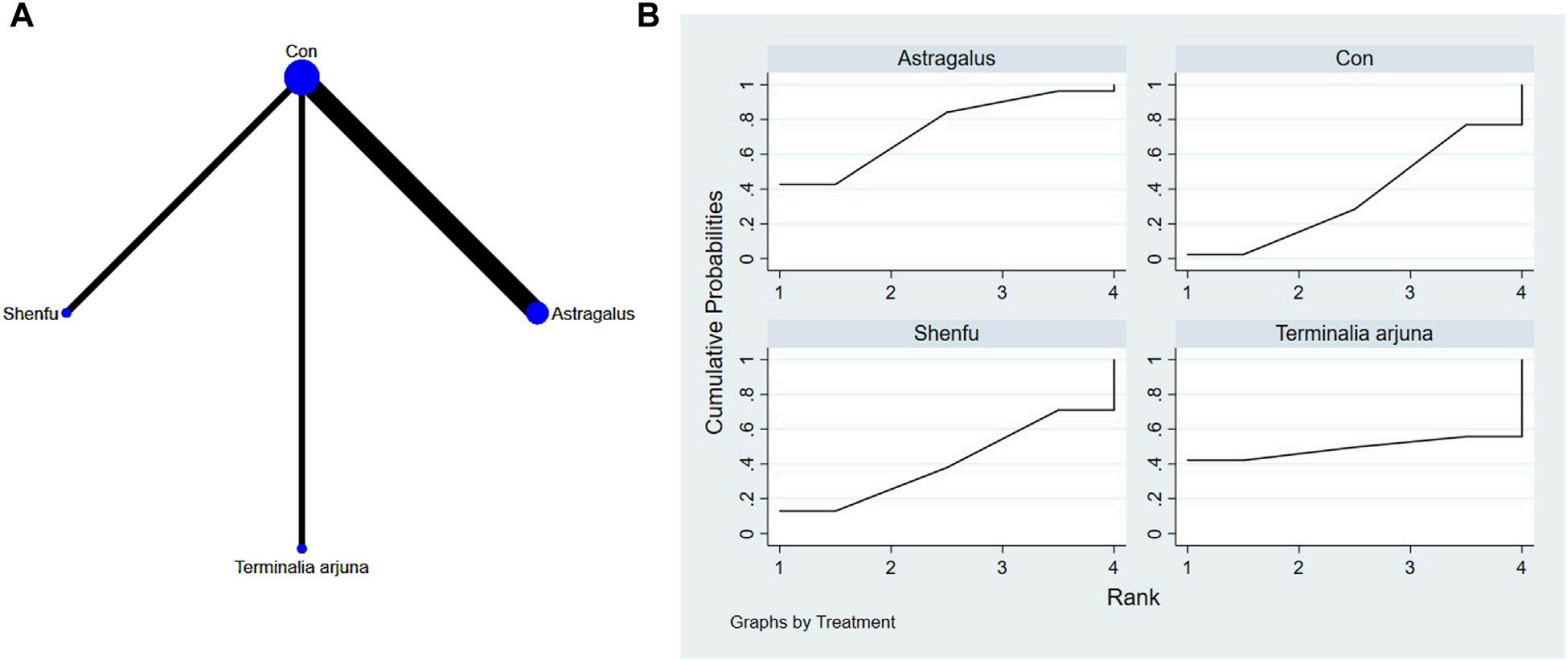
**(A)** NMA figure for *TNF-*α. **(B)** SUCRA plot for *TNF-*α.

**FIGURE 9:**
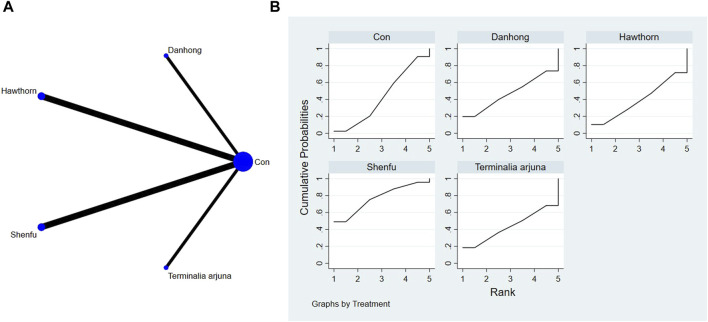
**(A)** NMA figure for QLQ. **(B)** SUCRA plot for QLQ.

The full NMA figure is shown in Figures 4A, 5A, 6A, 7A, 8A, 9A.

#### 3.4.1 Cardiac function indicators of 6-MWT

Consistency and inconsistency of *p*-values for both direct and indirect comparisons were assessed across all studies. With all *p*-values exceeding 0.05, the consistency effect among the studies was considered acceptable.

The findings from the network meta-analysis indicated that Shenfu extract [MD = −30.94, 95% CI = (5.14, 56.73)] demonstrated greater efficacy in enhancing the 6-minute walk test distance than the control group receiving hawthorn extract. Similarly, Shenfu extract [MD = −31.07, 95% CI = ((5.28, 56.87)] was found to be more effective than the control group receiving conventional treatment (CON). In the ranking of various plant extracts based on their probability of improving the 6-MWT distance, astragalus extract was ranked the highest according to the SUCRA (90.70%), followed by Shenfu extract (SUCRA: 71.6%), Danhong extract (SUCRA: 60.2%), *Terminalia arjuna* extract (SUCRA: 42.2%), hawthorn extract (SUCRA: 23.2%), and CON (SUCRA: 12.2%), as shown in Figure 4B and Table 3.

**TABLE 3:** League table on 6-MWT.

Astragalus	Shenfu	Danhong	*Terminalia arjuna*	Hawthorn	Con
Astragalus	−68.19 (−196.65, 60.28)	−76.76 (−204.94, 51.42)	−86.76 (−219.40, 45.88)	−99.12 (−224.96, 26.72)	−99.26 (−225.11, 26.59)
68.19 (−60.28, 196.65)	Shenfu	−8.57 (−44.05, 26.90)	−18.57 (−67.78, 30.63)	−30.94 (−56.73, -5.14)	−31.07 (−56.87, -5.28)
76.76 (−51.42, 204.94)	8.57 (−26.90, 44.05)	Danhong	−10.00 (−58.47, 38.47)	−22.36 (−46.71, 1.99)	−22.50 (−46.85, 1.85)
86.76 (−45.88, 219.40)	18.57 (−30.63, 67.78)	10.00 (−38.47, 58.47)	*Terminalia arjuna*	−12.36 (−54.27, 29.55)	−12.50 (−54.41, 29.41)
99.12 (−26.72, 224.96)	30.94 (5.14, 56.73)	22.36 (−1.99, 46.71)	12.36 (−29.55, 54.27)	Hawthorn	−0.14 (−0.49, 0.22)
99.26 (−26.59, 225.11)	31.07 (5.28, 56.87)	22.50 (−1.85, 46.85)	12.50 (−29.41, 54.41)	0.14 (−0.22, 0.49)	Con

#### 3.4.2 Cardiac function indicators of BNP

Consistency and inconsistency of *p*-values for both direct and indirect comparisons were assessed across all studies. With all *p*-values exceeding 0.05, the consistency effect among the studies was considered acceptable.

The network meta-analysis results indicated no significant differences in the league tables. In the ranking of various plant extracts based on their probability of reducing BNP levels, Shenfu extract was ranked the highest according to the SUCRA (68.2%), followed by *Terminalia arjuna* extract (SUCRA: 50.8%), hawthorn extract (SUCRA: 50.3%), Danhong extract (SUCRA: 40.8%), and CON (SUCRA: 39.9%), as shown in Figure 5B and Table 4.

**TABLE 4:** League table on BNP.

Shenfu	*Terminalia arjuna*	Hawthorn	Danhong	Con
Shenfu	66.38 (−773.34, 906.11)	76.88 (−387.78, 541.55)	126.57 (−328.22, 581.37)	114.88 (−140.86, 370.63)
−66.38 (−906.11, 773.34)	*Terminalia arjuna*	10.50 (−878.45, 899.45)	60.19 (−823.64, 944.02)	48.50 (−751.33, 848.33)
−76.88 (−541.55, 387.78)	−10.50 (−899.45, 878.45)	Hawthorn	49.69 (−490.63, 590.01)	38.00 (−349.96, 425.96)
−126.57 (−581.37, 328.22)	−60.19 (−944.02, 823.64)	−49.69 (−590.01, 490.63)	Danhong	−11.69 (−387.76, 364.38)
−114.88 (−370.63, 140.86)	−48.50 (−848.33, 751.33)	−38.00 (−425.96, 349.96)	11.69 (−364.38, 387.76)	Con

#### 3.4.3 Cardiac function indicators of LVEF

Consistency and inconsistency of *p*-values for both direct and indirect comparisons were assessed across all studies. With all *p*-values exceeding 0.05, the consistency effect among the studies was considered acceptable.

The network meta-analysis results indicated no significant differences in the league tables. In the ranking of various plant extracts based on their probability of improving the LVEF, red ginseng extract was ranked the highest according to the SUCRA (77.9%), followed by astragalus extract (SUCRA: 56.7%), hawthorn extract (SUCRA: 54.1%), Danhong extract (SUCRA: 47.6%), CON (SUCRA: 43.9%), *Terminalia arjuna* extract (SUCRA: 42.1%), and Shenfu extract (SUCRA: 27.6%), as shown in Figure 6B and Table 5.

**TABLE 5:** League table on LVEF.

Red ginseng	Astragalus	Hawthorn	Danhong	Con	*Terminalia arjuna*	Shenfu
Red ginseng	−11.37 (−44.35, 21.61)	−10.80 (−52.66, 31.06)	−13.80 (−55.36, 27.76)	−14.80 (−45.22, 15.62)	−16.00 (−57.60, 25.60)	−18.93 (−51.50, 13.64)
11.37 (−21.61, 44.35)	Astragalus	0.57 (−30.88, 32.02)	−2.43 (−33.49, 28.63)	−3.43 (−16.19, 9.32)	−4.63 (−35.74, 26.48)	−7.56 (−24.81, 9.69)
10.80 (−31.06, 52.66)	−0.57 (−32.02, 30.88)	Hawthorn	−3.00 (−43.36, 37.36)	−4.00 (−32.76, 24.75)	−5.20 (−45.60, 35.20)	−8.13 (−39.14, 22.89)
13.80 (−27.76, 55.36)	2.43 (−28.63, 33.49)	3.00 (−37.36, 43.36)	Danhong	−1.00 (−29.33, 27.32)	−2.20 (−42.30, 37.90)	−5.13 (−35.75, 25.49)
14.80 (−15.62, 45.22)	3.43 (−9.32, 16.19)	4.00 (−24.75, 32.76)	1.00 (−27.32, 29.33)	Con	−1.20 (−29.58, 27.18)	−4.12 (−15.76, 7.51)
16.00 (−25.60, 57.60)	4.63 (−26.48, 35.74)	5.20 (−35.20, 45.60)	2.20 (−37.90, 42.30)	1.20 (−27.18, 29.58)	*Terminalia arjuna*	−2.93 (−33.60, 27.74)
18.93 (−13.64, 51.50)	7.56 (−9.69, 24.81)	8.13 (−22.89, 39.14)	5.13 (−25.49, 35.75)	4.12 (−7.51, 15.76)	2.93 (−27.74, 33.60)	Shenfu

#### 3.4.4 Cardiac function indicators of NYHA

Consistency and inconsistency of *p*-values for both direct and indirect comparisons were assessed across all studies. With all *p*-values exceeding 0.05, the consistency effect among the studies was considered acceptable.

The findings from the network meta-analysis indicated that *Ginkgo biloba* extract [MD = −3.14, 95% CI =(1.06, 9.27)], astragalus extract [MD = −2.47, 95% CI =(1.35, 4.53)], and Shenfu extract [MD = 2.06, 95% CI =((1.44, 2.95)] were found to be more effective than the conventional treatment received by the control group. In the ranking of various plant extracts based on their probability of improving the NYHA, *Ginkgo biloba* extract was ranked the highest according to the SUCRA (76.5%), followed by astragalus extract (SUCRA: 67.8%), *Terminalia arjuna* extract (SUCRA: 64.2%), Shenfu extract (SUCRA: 56.6%), red ginseng extract (SUCRA: 50.7%), CON (SUCRA: 17.9%), and hawthorn extract (SUCRA: 16.4%), as shown in Figure 7B and Table 6.

**TABLE 6:** League table on NYHA.

*Ginkgo biloba*	Astragalus	*Terminalia arjuna*	Shenfu	Red ginseng	Con	Hawthorn
*Ginkgo biloba*	0.79 (0.23, 2.72)	0.97 (0.03, 29.24)	0.65 (0.21, 2.05)	0.58 (0.14, 2.41)	0.32 (0.11, 0.94)	0.17 (0.01, 2.36)
1.27 (0.37, 4.40)	Astragalus	1.24 (0.05, 32.99)	0.83 (0.41, 1.69)	0.74 (0.24, 2.23)	0.41 (0.22, 0.74)	0.21 (0.02, 2.57)
1.03 (0.03, 30.80)	0.81 (0.03, 21.45)	*Terminalia arjuna*	0.67 (0.03, 17.24)	0.59 (0.02, 17.02)	0.33 (0.01, 8.21)	0.17 (0.00, 9.61)
1.53 (0.49, 4.78)	1.20 (0.59, 2.43)	1.49 (0.06, 38.16)	Shenfu	0.88 (0.33, 2.39)	0.49 (0.34, 0.70)	0.25 (0.02, 2.94)
1.73 (0.42, 7.17)	1.36 (0.45, 4.10)	1.68 (0.06, 48.18)	1.13 (0.42, 3.05)	Red ginseng	0.55 (0.22, 1.39)	0.29 (0.02, 3.84)
3.14 (1.06, 9.27)	2.47 (1.35, 4.53)	3.06 (0.12, 76.95)	2.06 (1.44, 2.95)	1.82 (0.72, 4.59)	Con	0.52 (0.05, 5.89)
6.05 (0.42, 86.56)	4.76 (0.39, 58.20)	5.90 (0.10, 334.39)	3.97 (0.34, 46.25)	3.51 (0.26, 47.22)	1.93 (0.17, 21.89)	Hawthorn

#### 3.4.5 Cardiac function indicators of TNF-α

Consistency and inconsistency of *p*-values for both direct and indirect comparisons were assessed across all studies. With all *p*-values exceeding 0.05, the consistency effect among the studies was considered acceptable.

The network meta-analysis results indicated no significant differences in the league tables. In the ranking of various plant extracts based on their probability of reducing the TNF-α level, astragalus extract was ranked the highest according to the SUCRA (74.4%), followed by *Terminalia arjuna* extract (SUCRA:49.1%), Shenfu extract (SUCRA: 40.6%), and CON (SUCRA: 35.9%), as shown in Figure 8B and Table 7.

**TABLE 7:** League table on *TNF-*α.

Astragalus	*Terminalia arjuna*	Shenfu	Con
Astragalus	2.47 (−27.05, 32.00)	2.97 (−5.26, 11.20)	3.07 (−1.55, 7.69)
−2.47 (−32.00, 27.05)	*Terminalia arjuna*	0.50 (−29.45, 30.45)	0.60 (−28.56, 29.76)
−2.97 (−11.20, 5.26)	−0.50 (−30.45, 29.45)	Shenfu	0.10 (−6.71, 6.91)
−3.07 (−7.69, 1.55)	−0.60 (−29.76, 28.56)	−0.10 (−6.91, 6.71)	Con

#### 3.4.6 Cardiac function indicators of QLQ

Consistency and inconsistency of *p*-values for both direct and indirect comparisons were assessed across all studies. With all *p*-values exceeding 0.05, the consistency effect among the studies was considered acceptable.

The network meta-analysis results indicated no significant differences in the league tables. In the ranking of various plant extracts based on their probability of improving the QLQ, Shenfu extract was ranked the highest according to the SUCRA (77.0%), followed by Danhong extract (SUCRA: 47.1%), *Terminalia arjuna* extract (SUCRA: 43.4%), CON (SUCRA: 43.2%), and hawthorn extract (SUCRA: 39.3%), as shown in Figure 9B and Table 8.

**TABLE 8:** League table on QLQ.

Shenfu	Danhong	*Terminalia arjuna*	Con	Hawthorn
Shenfu	−4.57 (−20.36, 11.22)	−5.27 (−21.71, 11.16)	−4.87 (−14.20, 4.46)	−5.66 (−19.18, 7.86)
4.57 (−11.22, 20.36)	Danhong	−0.70 (−19.28, 17.88)	−0.30 (−13.04, 12.44)	−1.09 (−17.20, 15.02)
5.27 (−11.16, 21.71)	0.70 (−17.88, 19.28)	*Terminalia arjuna*	0.40 (−13.13, 13.93)	−0.39 (−17.14, 16.36)
4.87 (−4.46, 14.20)	0.30 (−12.44, 13.04)	−0.40 (−13.93, 13.13)	Con	−0.79 (−10.66, 9.08)
5.66 (−7.86, 19.18)	1.09 (−15.02, 17.20)	0.39 (−16.36, 17.14)	0.79 (−9.08, 10.66)	Hawthorn

### 3.5 Assessment of publication bias

**FIGURE 10:**
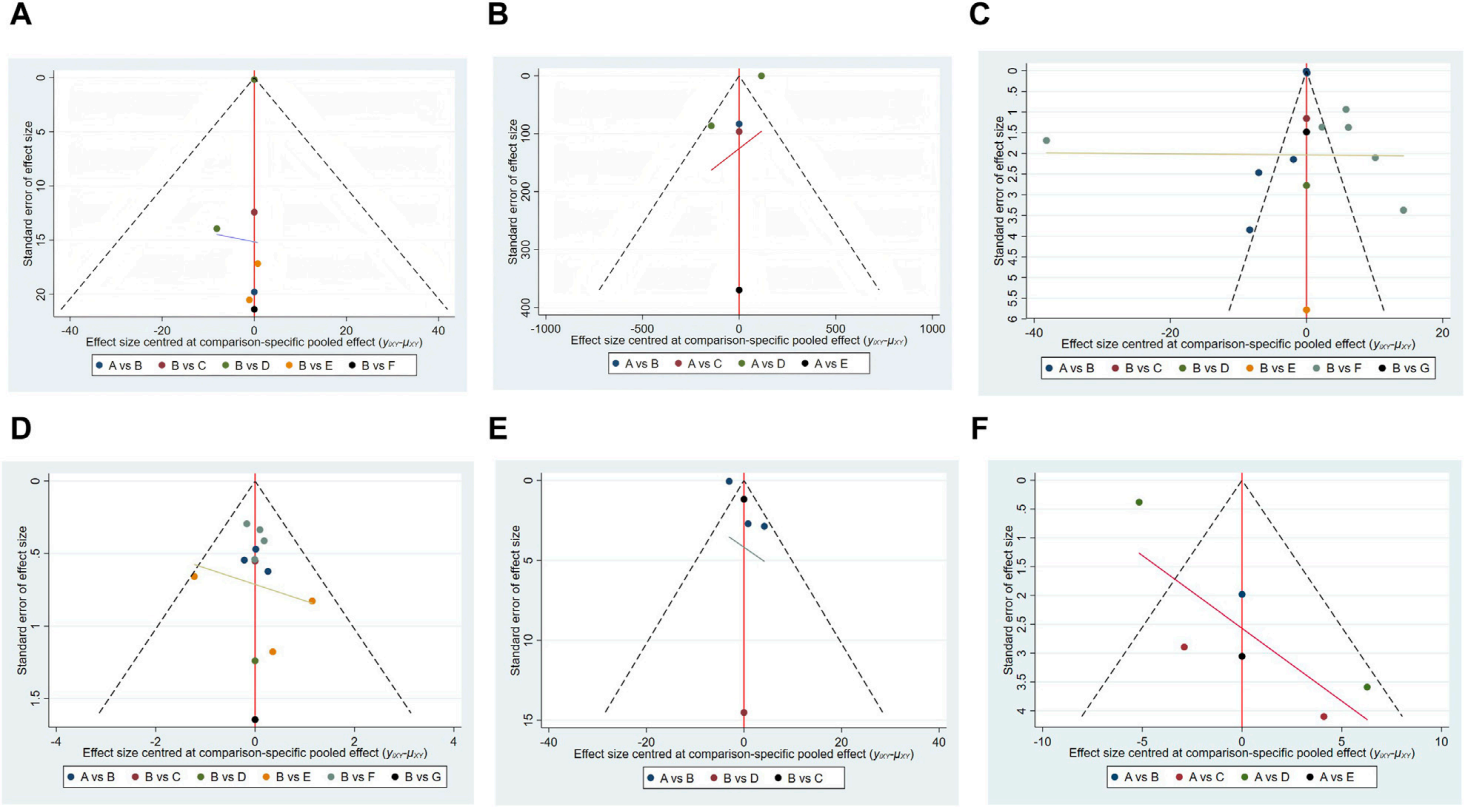
Funnel plot on publication bias. **(A)** 6-MWT; **(B)** BNP; **(C)** LVEF; **(D)** NYHA; **(E)** TNF-α; **(F)** QLQ.

A funnel plot is a common graphical test used to assess publication bias in meta-analyses (Egger et al., 1997). Separate funnel plots were developed for all outcome indicators to assess the possibility of publication bias, as shown in Figure 10.

## 4 Discussion

In this study, we conducted a comparative analysis of the efficacy of various plant extracts in enhancing the clinical parameters in patients with heart failure. A total of 20 studies were included, encompassing 7 distinct plant extracts and involving 2,077 patients diagnosed with heart failure. Our findings indicated that astragalus extract was the most effective in improving 6-MWT scores and reducing TNF-α levels. Shenfu extract demonstrated superior efficacy in decreasing BNP values and enhancing quality of life. Red ginseng extract yielded better outcomes in terms of improving LVEF, while *Ginkgo biloba* extract was more effective in enhancing cardiac function class.

In our study, Shenfu injection demonstrated efficacy in enhancing quality of life, reducing BNP levels, decreasing TNF-α, and improving cardiac function grading. HF represents a significant cause of morbidity and mortality globally. Natriuretic peptides (NPs) are cardioprotective hormones secreted by cardiomyocytes in response to pressure or volume overload (Goetze et al., 2020). Both BNP and N-terminal pro-B-type natriuretic peptide (NT-proBNP) have been extensively shown to contribute to the diagnosis and risk stratification of HF, and these biomarkers are increasingly utilized as tools for population screening (Castiglione et al., 2022). Given that heart failure can profoundly impact patients’ quality of life, enhancing it is a critical objective in heart failure management. Shenfu injection (SFI) is a herbal therapeutic approach for heart failure. Nonetheless, the precise mechanisms by which ginseng exerts its therapeutic effects in heart failure remain inadequately understood. Li et al. (2024) utilized subcutaneous multipoint injection of isoprenaline (ISO) to develop a heart failure model. Their findings suggest that Shenfu injection may effectively enhance cardiac function in rats with ISO-induced heart failure, potentially through the modulation of the pro-/anti-inflammatory balance and the reduction of serum and urinary trimethylamine N-oxide (TMAO) levels (Li et al., 2024). Additionally, Shenfu soup has been shown to enhance cardiac contractility, increase coronary blood supply, improve ischemic myocardial metabolism, scavenge free radicals, and protect myocardial ultrastructure (Wei et al., 2015). Zhou et al. (2023) demonstrated that ginseng and sorrel injection can significantly improve the quality of life and exercise tolerance in patients with chronic heart failure, and it can reduce blood levels of BNP/NT-proBNP (Zhou et al., 2023).

Red ginseng extract serves as the primary component of Shenmai injection (SMI). In our study, extracts of red ginseng, astragalus, and hawthorn demonstrated significant efficacy in enhancing LVEF, with red ginseng extract exhibiting the most pronounced effect. Heart failure is a clinical syndrome characterized by dyspnea or exercise intolerance resulting from impaired ventricular filling, ejection, or both. The diagnosis is primarily based on elevated natriuretic peptide levels above age- and context-specific thresholds, alongside the identification of left ventricular systolic dysfunction, as indicated by echocardiographic measurement of an LVEF of 40% or less (Murphy et al., 2020). Wu et al. (2023) demonstrated that Shenmai injection enhances cardiac function in patients with congestive heart failure (CHF) through its anti-apoptotic, antioxidant, and anti-inflammatory properties, along with improving myocardial metabolism. Similarly, Zheng et al. (2011) reported that SMI leads to improvements in cardiac index and myocardial contractility, with some amelioration of heart failure symptoms. Yang et al. (2024) found that SMI facilitates myocardial lipid metabolism and mitigates heart failure following myocardial infarction, as evidenced by a post-infarction heart failure mouse model and an ischemia/reperfusion (I/R) cell model. Furthermore, another animal study indicated that SMI promotes angiogenesis and cardiac remodeling, resulting in enhanced LVEF in a rat model of myocardial ischemia/reperfusion injury (MIRI) (Liu et al., 2018). Myocardial fibrosis, a primary pathological feature of hypertensive heart failure, is reportedly alleviated by Ginseng-Mai injection, which effectively improves heart failure by inhibiting myocardial fibrosis (Hu et al., 2023).

In the present study, astragalus extract demonstrated significant efficacy in enhancing the 6-min walking distance and reducing TNF-α levels. Additionally, it proved effective in improving LVEF and the NYHA classification. Inflammatory cytokines are persistently elevated in patients with congestive heart failure, with TNF-α being a pivotal pro-inflammatory cytokine that exacerbates heart failure by counteracting the anti-inflammatory response and disrupting systemic homeostasis (Schumacher and Naga Prasad, 2018). The 6-minute walk test is a widely utilized and well-tolerated assessment tool for evaluating the functional capacity of heart failure patients, offering reliable insights into daily activities and short-term prognosis, particularly in those with heart failure and reduced ejection fraction (Giannitsi et al., 2019). Astragalus polysaccharide (APS) is a bioactive compound derived from *Astragalus membranaceus*. The studies have elucidated the bioactivity, potential targets, and molecular mechanisms of APS in the context of heart failure. These studies suggest that isorhamnetin, quercetin, trichothecenes, spinosyn, and kaempferol are likely the principal active components of APS, exerting cardioprotective effects by modulating the expression of the estrogen receptor (ESR1) (Chen et al., 2024). Ma et al. (2023) demonstrated that APS mitigates heart failure-induced cachexia by reducing excessive lipid consumption in both white and brown adipose tissues. Astragaloside IV (AS-IV), the primary active constituent of astragalus, has been utilized as a therapeutic agent for heart failure. It is proposed that AS-IV influences the HSF1/VEGF signaling pathway, enhances angiogenesis, and alleviates pressure overload-induced heart failure (Du et al., 2024). In a clinical study involving 43 patients with acute myocardial infarction receiving astragalus, significant improvements in left ventricular function were observed. The antioxidant properties of astragalus may contribute to its cardiotonic effects (Chen et al., 1995).

The NYHA classification serves as a crucial instrument for the risk stratification of HF. It has been demonstrated to have a strong correlation with survival rates and is utilized to indicate the severity of acute heart failure (AHF) (Blacher et al., 2021). In the current study, *Ginkgo biloba* extract exhibited significant efficacy in improving the NYHA classification. The beneficial effects of *Ginkgo biloba* extracts are primarily attributed to their high concentrations of flavonoids and terpenoids, antioxidant properties (Karmazyn and Gan, 2023), ability to reduce reperfusion-induced arrhythmias (Tosaki et al., 1993), mitigate ischemia–reperfusion injury, and promote functional recovery (Tosaki et al., 1994; Tosaki et al., 1996). Evidence suggests that *Ginkgo biloba* extract exerts a protective effect against adriamycin-induced cardiotoxicity, potentially through mechanisms involving the inhibition of mitochondria-dependent pro-apoptotic signaling and the reduction of pro-inflammatory factors (Liu et al., 2008). Due to its anti-inflammatory properties, *Ginkgo biloba* extract has been further explored for its potential therapeutic benefits in cardiac pathology secondary to viral myocarditis (Wang W. et al., 2019).

The present study focuses on evaluating the efficacy rankings of various botanical extracts (including oral preparations and injectable formulations) across different indicators of heart failure. Consistent with previous research findings, Chinese patent medicines have demonstrated significant efficacy in the treatment of heart failure. Lin et al. (2021) demonstrated that among Chinese patent medicines (injections) combined with conventional therapy, Xinmailong was the most effective in improving the NYHA functional classification efficiency and reducing BNP levels; Shenmai was the most effective in the 6-minute walk test; YiQiFuMai lyophilized injection was the most effective for left ventricular ejection fraction; Huangqi was the most effective in improving stroke volume; and Shenfu performed the best in enhancing quality of life. Guo et al. (2023) showed that among Chinese patent medicines (oral preparations) combined with conventional therapy, Shexiang Baoxin Pill was the most effective in improving the NYHA functional classification efficiency, Tongxinluo capsule was the most effective in ameliorating the ratio of early-to-late diastolic mitral inflow velocity (E/A ratio), and Qili Qiangxin was the most effective in reducing NT-proBNP levels and improving the 6-minute walk test results. Comparative studies demonstrate that Huangqi exhibits stable and remarkable efficacy in improving patients’ exercise endurance, while Shenfu preparations show unique advantages in enhancing subjective well-being and quality of life. However, discrepancies exist in research conclusions due to variations in intervention methods (such as single plant extracts versus compound formulations, drug dosage forms, and administration routes) and study populations. Overall, current research aligns closely with previous studies in establishing evidence-based systems for TCM treatment of heart failure, with differences reflecting the complexity of heart failure management and the need for personalized approaches. Future research should provide detailed reports on intervention details (e.g., standardization levels of extracts, injectable manufacturers, and oral dosage forms) and conduct subgroup analyses to reduce heterogeneity and offer more precise clinical guidance.

## 5 Strengths and limitations

Our study incorporated 20 studies encompassing a total of 2,077 patients, representing a substantial sample size. The systematic review adhered to a rigorous screening process, used advanced statistical methodologies, and integrated data from analogous studies to mitigate random errors. A thorough quality assessment of the included studies was conducted to align the results more closely with real-world conditions, thereby providing reliable conclusions for both clinicians and patients. Additionally, the study synthesized numerous and varied plant extracts, selecting several commonly used extracts to inform clinical decision-making. Nevertheless, variations in patient characteristics, interventions, and other study parameters, such as the dosage discrepancies of identical plant extracts, may impact the stability and interpretation of the results. Although statistical methods can partially adjust for these differences, they cannot completely eliminate the effects of heterogeneity. Furthermore, the quality of the original studies included may be inconsistent, with some studies exhibiting small sample sizes and suboptimal design, which could affect the overall quality of the evidence.

## 6 Conclusion

Astragalus extract, red ginseng extract, *Ginkgo biloba* extract, *Terminalia arjuna* extract, and Shenfu extract have demonstrated significant efficacy in heart failure management for the selection of optimal plant extracts based on enhanced indicators of cardiac function outcomes. Continued research through rigorous randomized controlled trials is essential to substantiate and refine the current evidence.

## Data Availability

The original contributions presented in the study are included in the article/Supplementary Material, further inquiries can be directed to the corresponding authors.
