# Supplementary material for: Effects of plant extracts on patients with heart failure: a network meta-analysis of randomized controlled trials
Source: Front Pharmacol. 2025 Nov 5;16:1648811. doi: 10.3389/fphar.2025.1648811 (PMC12626924; doi:10.3389/fphar.2025.1648811)
Supplement: Supplementary file 1 [file DataSheet1.pdf]

The pie charts illustrate the number and proportion of randomized controlled trials (RCTs) included in the network meta-analysis across different plant extract intervention groups (Figures 4A, 5A, 6A, 7A, 8A, 9A).

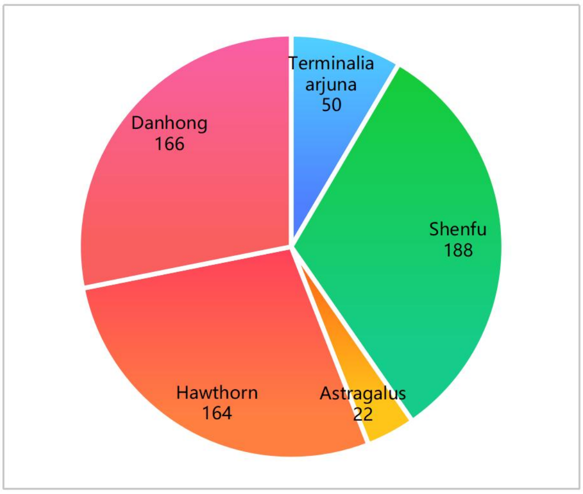

Figure 4A. This pie chart shows the number and proportion of included RCTs across different plant extract intervention groups in improving the 6-MWT.

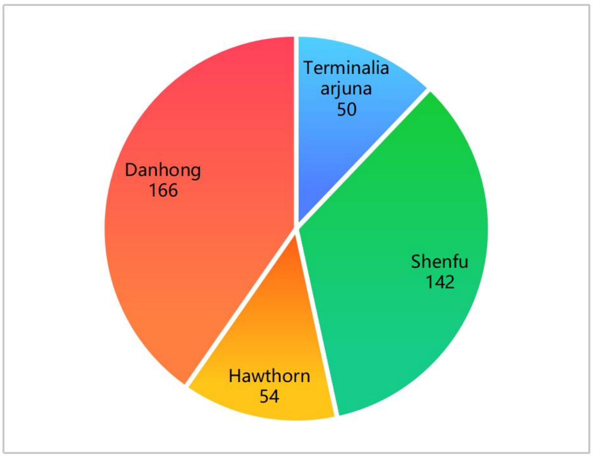

Figure 5A. This pie chart shows the number and proportion of included RCTs across different plant extract intervention groups in improving the BNP.

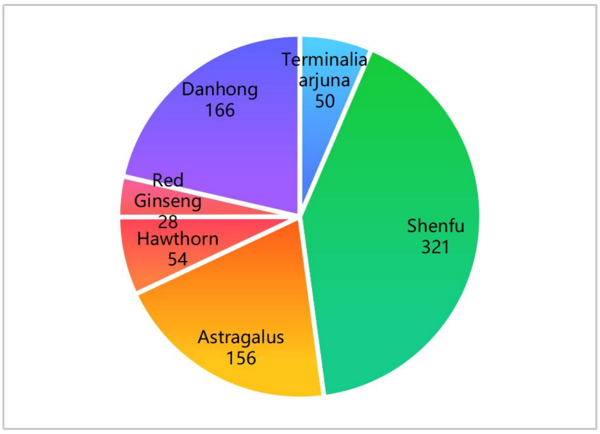

Figure 6A. This pie chart shows the number and proportion of included RCTs across different plant extract intervention groups in improving the LVEF

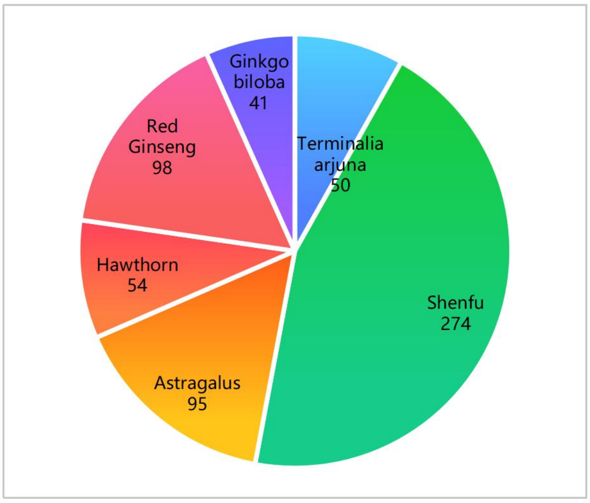

Figure 7A. This pie chart shows the number and proportion of included RCTs across different plant extract intervention groups in improving the NYHA.

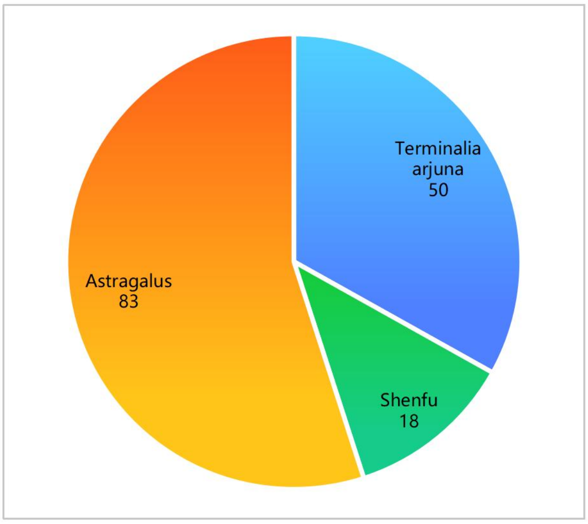

Figure 8A. This pie chart shows the number and proportion of included RCTs across different plant extract intervention groups in improving the TNF-a.

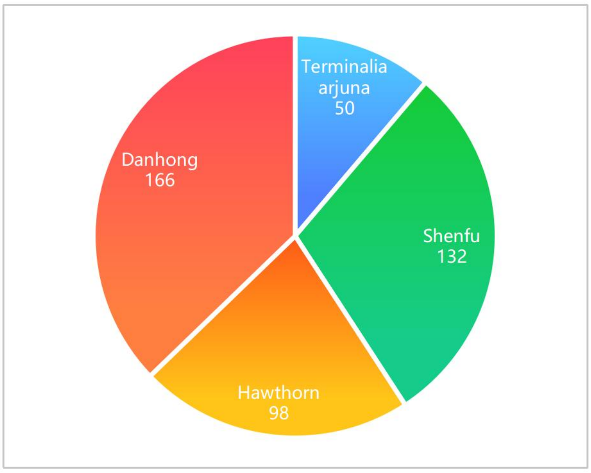

Figure 9A. This pie chart shows the number and proportion of included RCTs across different plant extract intervention groups in improving the QLQ.
